# A Comprehensive Analysis of RALF Proteins in Green Plants Suggests There Are Two Distinct Functional Groups

**DOI:** 10.3389/fpls.2017.00037

**Published:** 2017-01-24

**Authors:** Liam Campbell, Simon R. Turner

**Affiliations:** Faculty of Biology, Medicine and Health, School of Biological Science, University of ManchesterManchester, UK

**Keywords:** RALF, peptide, development, phylogeny, growth, evolution

## Abstract

Rapid Alkalinization Factors (RALFs) are small, cysteine-rich peptides known to be involved in various aspects of plant development and growth. Although RALF peptides have been identified within many species, a single wide-ranging phylogenetic analysis of the family across the plant kingdom has not yet been undertaken. Here, we identified RALF proteins from 51 plant species that represent a variety of land plant lineages. The inferred evolutionary history of the 795 identified RALFs suggests that the family has diverged into four major clades. We found that much of the variation across the family exists within the mature peptide region, suggesting clade-specific functional diversification. Clades I, II, and III contain the features that have been identified as important for RALF activity, including the RRXL cleavage site and the YISY motif required for receptor binding. In contrast, members of clades IV that represent a third of the total dataset, is highly diverged and lacks these features that are typical of RALFs. Members of clade IV also exhibit distinct expression patterns and physico-chemical properties. These differences suggest a functional divergence of clades and consequently, we propose that the peptides within clade IV are not true RALFs, but are more accurately described as RALF-related peptides. Expansion of this RALF–related clade in the Brassicaceae is responsible for the large number of RALF genes that have been previously described in *Arabidopsis thaliana*. Future experimental work will help to establish the nature of the relationship between the true RALFs and the RALF-related peptides, and whether they function in a similar manner.

## Introduction

Complex, multicellular organisms such as plants use short- and long-distance signaling networks to allow for communication across different regions of the organism. Although this signaling is fundamentally important during plant growth, these networks also function to coordinate systemic responses to environmental stimuli. In recent years, it has emerged that small secreted peptides are a key component of these networks, controlling many critical processes, including the regulation of stem cell division and differentiation, cell expansion, stomatal development and gravitropism (reviewed by Czyzewicz et al., 2013; Delay et al., 2013; Matsubayashi, 2014). One such small peptide family are the Rapid Alkalinization Factors (RALFs), first discovered through their ability to rapidly alkalinize tobacco cell cultures (Pearce et al., 2001). The canonical peptide, RALF1, was found to arrest tomato and Arabidopsis root growth with no activation of defense pathways (Pearce et al., 2001), initially suggesting a developmental role for these peptides. However, subsequent studies have identified a wide variety of roles for members of the RALF family, including cell expansion (Haruta et al., 2014), lateral root development (Murphy et al., 2016), root hair growth (Wu et al., 2007), pollen tube elongation (Covey et al., 2010), as well as stress (Atkinson et al., 2013). The diversity of these roles indicates that RALF peptides are fundamentally important for plant development.

RALF proteins are cysteine-rich and typically have a full length of 80–120 amino acids. They are translated as a preproprotein containing an N-terminal signal peptide that leads to their secretion and a C-terminal mature peptide with four di-sulfide bond-forming cysteine residues (Pearce et al., 2001, 2010). AtRALF23 was found to be cleaved at a di-basic RRXL site by the serine protease SITE-1 PROTEASE (AtSIP1), and this processing is essential for proper functioning of the peptide (Srivastava et al., 2009). Until recently, the downstream mechanisms through which RALFs function was unknown, although a conserved YISY motif was known to be required for AtRALF1-receptor binding (Pearce et al., 2010). FERONIA (FER), a receptor-kinase from the *Catharantus roseus* RLK1-like (CrRLK1L) subfamily (Lindner et al., 2012), has been identified as a receptor for AtRALF1 and is involved in the rapid alkalization response (Haruta et al., 2014). Knock-out alleles of *fer*, resulting from T-DNA insertion, are insensitive to AtRALF1 treatment. However, co-immunoprecipitation experiments suggest that AtRALF1 also binds to other receptors (Haruta et al., 2014). Whether FER acts as a receptor for other RALFs is currently not clear, and no other RALF receptors have been identified to date. Very recently, the receptor-like cytoplasmic RPM1-induced protein kinase (RIPK) has been identified as an intracellular, interacting partner that is directly phosphorylated by FER and is crucial for the relaying of the RALF1-FER signal (Du et al., 2016).

Consistent with their diverse roles in development and stress responses, RALFs have so far been identified in a variety of species, including monocots, eudicots and early-diverging lineages (Pearce et al., 2001; Haruta and Constabel, 2003; Germain et al., 2005; Silverstein et al., 2007; Cao and Shi, 2012; Ghorbani et al., 2015; Sharma et al., 2016). A duplication analysis has previously found that a large percentage of plant RALF proteins have evolved through tandem duplication, with this being responsible for the varying numbers of RALF proteins within previously investigated plant species (Cao and Shi, 2012). This is further demonstrated by the presence of pairs of RALF proteins exhibiting high homology to one another (Cao and Shi, 2012). Intriguingly, biologically-active RALF homologs, typically of RALF1, have also been identified within numerous fungal phytopathogens, with these potentially acting in plant-pathogen interactions (Thynne et al., 2016). The seemingly ubiquitous presence of RALFs across the plant kingdom is further evidence of their general importance. However, no single wide-ranging, species-rich phylogenetic study of the RALF family has yet been undertaken. In an attempt to uncover new insights into the evolutionary history of the RALF family, we here present a comprehensive identification and analysis of RALF proteins from more than 50 diverse green plant proteomes obtained in a consistent format from Phytozome (Goodstein et al., 2012). We reveal that RALFs have diverged into distinct groups, each containing identifiable differences in amino acid sequence. Importantly, much of this sequence diversification exists within the mature peptide region and is likely to define receptor binding and hence biological activity. One of the identified clades, which represents a third of all identified proteins, lacks many typical RALF features. We propose that these do not represent true RALFs and should be considered independently to the more typical RALF proteins.

## Materials and methods

### Identification of RALF proteins

Conserved motifs in RALF proteins (Figure S1) were identified using MEME (Bailey and Elkan, 1994) using 37 previously identified RALFS as a query (Cao and Shi, 2012). These motifs were identified in other plant species by scanning with FIMO (Grant et al., 2011). Proteomes for the following species were retrieved from Phytozome v11.0 (Goodstein et al., 2012): *Amaranthus hypochondriacus, Amborella trichopoda, Ananas comosus, Aquilegia coerulea, Arabidopsis halleri, Arabidopsis lyrata, Arabidopsis thaliana, Brachypodium distachyon, Brachypodium stacei, Brassica rapa, Capsella grandiflora, Capsella rubella, Carica papaya, Chlamydomonas reinhardtii, Citrus clementina, Citrus sinensis, Coccomyxa subellipsoidea C-169, Cucumis sativus, Eucalyptus grandis, Eutrema salsugineum, Fragaria vesca, Glycine max, Gossypium raimondii, Linum usitatissimum, Malus domestica, Medicago truncatula, Micromonas pusilla, Mimulus guttatus, Musa acuminate, Oryza sativa, Ostreococcus lucimarinus, Panicum hallii, Panicum virgatum, Phaseolus vulgaris, Physcomitrella patens, Populus trichocarpa, Prunus persica, Ricinus communis, Salix purpurea, Selaginella moellendorffii, Setaria italic, Setaria viridis, Solanum lycopersicum, Solanum tuberosum, Sorghum bicolor, Spirodela polyrhiza, Theobroma cacao, Vitis vinifera, Volvox carteri, Zea mays, and Zostera marina*. Genomic data such as genome size and total gene number were extracted from the relevant publication for each species (see Table S1). A stringent *q*-value cut off of 0.05 was used to remove low-scoring hits to the six motifs identified by MEME.

### Construction of RALF alignments and phylogenetic trees

All protein alignments were initially created using the MUSCLE algorithm (Edgar, 2004) within the AliView alignment editor (Larsson, 2014). This was followed by manual optimisation to improve the alignment in regions that had been clearly misaligned by MUSCLE. Approximately-maximum likelihood phylogenetic trees were created for full-length proteins and mature peptides using FastTree v2.1 (Price et al., 2010), with 4 rounds of minimum-evolution SPR moves (option: -spr 4) and exhaustive nearest-neighbor interchanges (options: -mlacc 2 -slownni) to improve accuracy. All other parameters were left as default, including the calculation of local-support values by the Shimodaira-Hasegawa test (Shimodaira and Hasegawa, 1999). The inferred phyloXML trees were viewed in Archaeopteryx v0.9916 (Han and Zmasek, 2009) to identify RALF clades and sub-clades. WebLogo3 (Crooks et al., 2004) (http://weblogo.threeplusone.com/) was used to provide a visual summary of conserved residues within the alignments. The inferred phylogenetic trees have been uploaded to the TreeBASE repository (Piel et al., 2002) and can be accessed at http://purl.org/phylo/treebase/phylows/study/TB2:S20366.

### CLANS pairwise-similarity plots

To perform pairwise BLAST (Altschul et al., 1990) hits between each individual protein, full-length unaligned sequences of all 795 RALFs were uploaded to the online CLANS server (Frickey and Lupas, 2004) that is a part of the MPI Bioinformatics Toolkit (Alva et al., 2016). The BLOSUM80 substitution matrix was used for scoring and the HSP option was enabled. The output from this server was used within the standalone CLANS software to create the 2D similarity plots. A *p*-value threshold of 1e^−10^ was used to remove low-scoring BLAST searches and a minimum attraction value of 50 was used to restrict the proteins to within a reasonable 2D space. As CLANS is non-deterministic, we ran the analysis many times, each from different initialization states, to confirm that the separation of the proteins was reproducible and consistent. We stopped each analysis when the proteins had become stationary within the 2D plot.

### Protein physico-chemical property prediction

The online multi-cleverMachine tool (Klus et al., 2014) was used to compare the physico-chemical properties of RALF proteins. Unaligned sequences for each clade were uploaded to the server in FASTA format, with “clade IV” proteins being denoted as the positive set and clade I, II, and III proteins as negative sets. Ten scales were used to predict each property and detailed statistical comparisons were viewed using the boxplot and ROC curve functions found within the output screen.

### Expression analysis

The mRNA expression of *A. thaliana* and *Z. mays* RALF genes was analyzed across publically-available RNAseq datasets that are included within Genevestigator (Hruz et al., 2008). A total of 1031 samples across all datasets were analyzed. A clustered heat-map representing the log-2 absolute expression values throughout the anatomy of the plant was obtained using the “Hierarchical Clustering” tool within Genevestigator, with both the genes and conditions subjected to Euclidian-distance clustering.

## Results

### Identification of RALF proteins from 51 plant genomes

Previous genome-wide identifications and phylogenetic analyses of the RALF family have focused on either six (Cao and Shi, 2012) or four (Sharma et al., 2016) plant species. We sought to broaden this range by taking advantage of the Phytozome v11.0 genomic resource (Goodstein et al., 2012), which provides comprehensive sequence data and accompanying annotation for a variety of green plant species. A full list of the 51 species included in this study can be found in Table 1. This diverse range of species includes lineages that have been excluded from previous RALF phylogenetic analyses, such as the Rosaceae, and generally allows for a more informative analysis with greater resolution. Additionally, though a small number of RALFs have been previously found in plants such as potato (Germain et al., 2005) and *M. truncatula* (Pearce et al., 2001; Combier et al., 2008), these studies were performed before a full genome was available, and were instead reliant upon expressed sequence tags (ESTs). Therefore, revisiting such species in light of the numerous full plant genomes now available should allow for a more accurate representation of the RALF family.

**Table 1:** **The species analyzed within this study**.

**Species**	**Order**	**Family**	**Total**	**Clade counts**			
				**1**	**2**	**3**	**4**
**EUDICOTS**							
*Amaranthus hypochondriacus*	Caryophyllales	Amaranthaceae	12	2	4	4	2
*Aquilegia coerulea*	Ranunculales	Ranunculaceae	12	1	3	3	5
*Arabidopsis halleri*	Brassicales	Brassicaceae	25	3	1	5	16
*Arabidopsis lyrata*	Brassicales	Brassicaceae	33	4	2	7	20
*Arabidopsis thaliana*	Brassicales	Brassicaceae	37	3	3	8	23
*Brassica rapa*	Brassicales	Brassicaceae	32	6	5	12	9
*Capsella grandiflora*	Brassicales	Brassicaceae	24	3	2	7	12
*Capsella rubella*	Brassicales	Brassicaceae	33	5	2	8	18
*Carica papaya*	Brassicales	Caricaceae	17	1	4	2	11
*Citrus clementina*			13	3	5	5	1
*Citrus sinensis*	Sapindales	Rutaceae	14	1	5	7	1
*Cucumis sativus*	Cucurbitales	Cucurbitaceae	13	2	6	4	3
*Eucalyptus grandis*	Myrtales	Myrtaceae	16	2	4	5	5
*Eutrema salsugineum*	Brassicales	Brassicaceae	35	4	2	10	19
*Fragaria vesca*	Rosales	Rosaceae	9	1	4	3	1
*Glycine max*	Fabales	Fabaceae	23	2	8	11	2
*Gossypium raimondii*	Malvales	Malvaceae	33	0	10	12	11
*Linum usitatissimum*	Malpighiales	Linaceae	20	0	7	8	5
*Malus domestica*	Rosales	Rosaceae	33	2	14	11	6
*Medicago truncatula*	Fabales	Fabaceae	13	0	4	4	5
*Mimulus guttatus*	Lamiales	Phrymaceae	17	0	6	5	4
*Phaseolus vulgaris*	Fabales	Fabaceae	9	1	4	3	1
*Populus trichocarpa*	Malpighiales	Salicaceae	20	2	6	7	5
*Prunus persica*	Rosales	Rosaceae	13	1	5	4	3
*Ricinus communis*	Malpighiales	Euphorbiaceae	18	0	5	4	9
*Salix purpurea*	Malpighiales	Salicaceae	32	4	10	7	11
*Solanum lycopersicum*	Solanales	Solanaceae	8	1	2	4	1
*Solanum tuberosum*	Solanales	Solanaceae	16	1	5	6	4
*Theobroma cacao*	Malvales	Malvaceae	13	0	5	5	3
*Vitis vinifera*	Vitales	Vitaceae	4	0	3	1	0
**MONOCOTS**							
*Ananas comosus*	Poales	Bromeliaceae	14	0	1	10	3
*Brachypodium distachyon*	Poales	Poaceae	10	0	0	9	1
*Brachypodium stacei*	Poales	Poaceae	11	0	0	10	1
*Musa acuminata*	Zingiberales	Musaceae	13	7	0	6	0
*Oryza sativa*	Poales	Poaceae	14	0	0	13	1
*Panicum hallii*	Poales	Poaceae	13	0	0	8	5
*Panicum virgatum*	Poales	Poaceae	31	0	0	22	9
*Setaria italica*	Poales	Poaceae	15	0	0	9	6
*Setaria viridis*	Poales	Poaceae	15	0	0	9	6
*Sorghum bicolor*	Poales	Poaceae	16	0	0	11	5
*Spirodela polyrhiza*	Alismatales	Araceae	7	0	1	3	3
*Zea mays*	Poales	Poaceae	20	0	0	15	5
*Zostera marina*	Alismatales	Zosteraceae	7	0	2	5	0
**EARLY-DIVERGING ANGIOSPERM**							
*Amborella trichopoda*			9	0	1	5	3
**EARLY-DIVERGING PLANTS**							
*Physcomitrella patens*			2	0	0	2	0
*Selaginella moellendorffii*			1	0	0	1	0
**CHLOROPHYTES**							
*Chlamydomonas reinhardtii*			0	0	0	0	0
*Coccomyxa subellipsoidea C-169*			0	0	0	0	0
*Micromonas pusilla*			0	0	0	0	0
*Ostreococcus lucimarinus*			0	0	0	0	0
*Volvox carteri*			0	0	0	0	0

*The total number of identified RALFs and the number found within each clade are shown*.

To find RALF proteins across the 51 included species, MEME (Bailey and Elkan, 1994) was first used to detect up to six conserved amino acid motifs within the previously identified 37 *A. thaliana* RALFs (Cao and Shi, 2012; Figure S1). This was followed by the identification of regions matching to these motifs across the proteome of all 51 species using FIMO (Grant et al., 2011). We discovered a total of 795 RALFs, with a breakdown of the number per species shown in Table 1. None of the analyzed proteomes were found to contain more than the 37 RALFs identified in *A. thaliana*, though there are other eudicot species with large numbers of RALFs, such as the 33 found in the apple (*M. domestica*) proteome. In general, the eudicots contain more RALF proteins on average (~20) than the monocots (~14), however, the angiosperm with the fewest number of RALFs is the early-diverging rosid domesticated grape (*V. vinifera*), which only has four RALF proteins.

### Rapid expansion of the RALF family in the Brassicaceae

**Figure 1:**
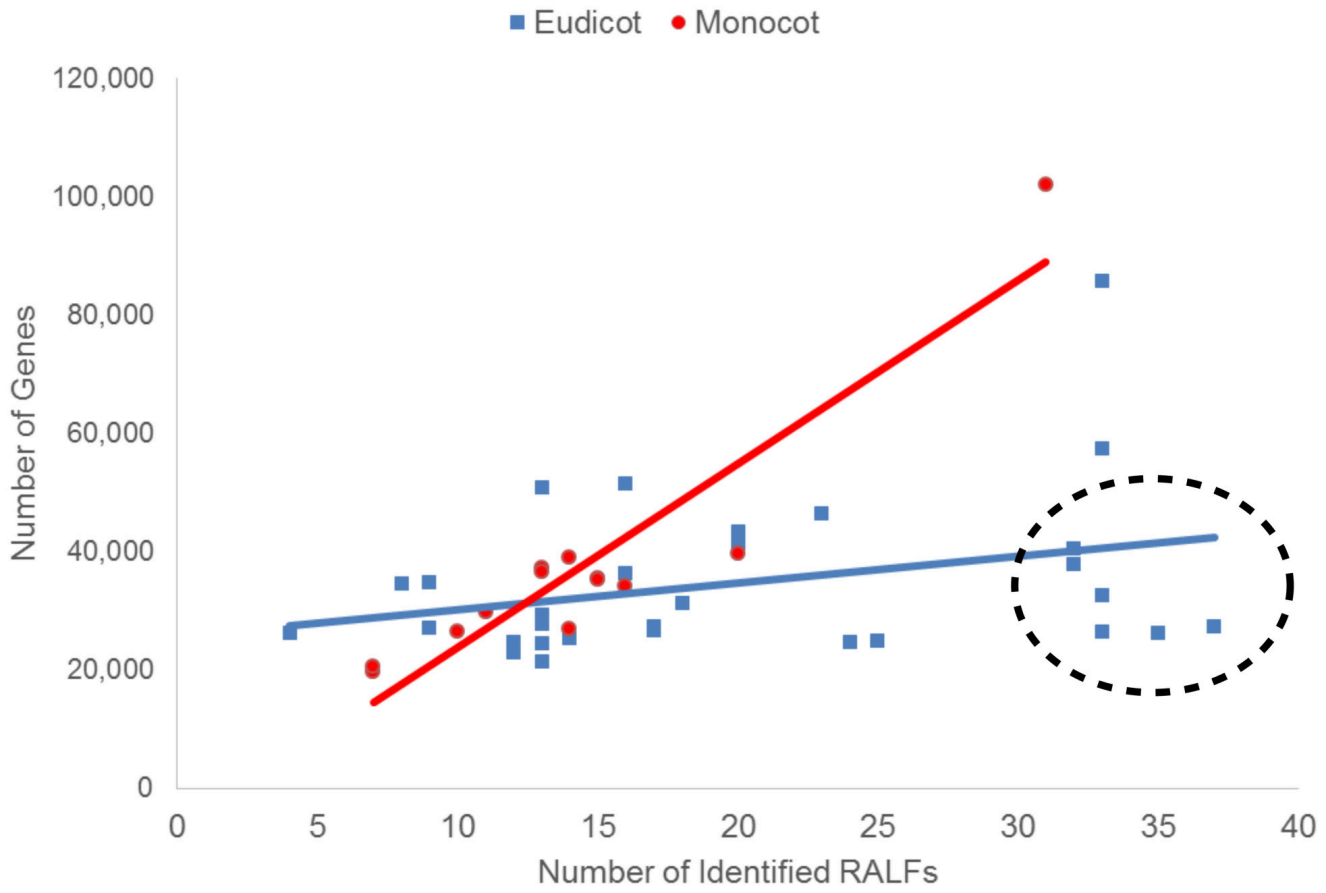
**The relationship between the total number of genes within the genome and the number of identified RALFs for the monocots (red) and eudicots (blue)**. Species with unusually high numbers of RALFs based upon their genome content are circled.

The difference in the average number of RALF genes between the monocots and eudicots could mean that the genetic mechanisms underlying the evolution of the RALF family was distinct in each group. On the other hand, such differences may simply be a consequence of the eudicots analyzed having larger genomes than the monocots. To investigate this, for each species we compared the number of RALF genes to the total number of genes in the genome (Figure 1), genome size (Mbp; Figure S2), and gene density (genes/Mbp). We found a strong positive correlation (*r* = 0.66) between the number of RALFs and genome size for monocots, but a weak negative correlation (*r* = −0.13) for eudicots. Furthermore, the number of RALF genes correlates very strongly (*r* = 0.93) with the total number of genes across the monocots, but there is only a weak correlation (*r* = 0.32) for the eudicots. This data suggests that in monocots, RALF diversification has occurred at a very consistent rate that is proportional to overall changes in genome size, such as those caused by genome duplications. In eudicots however, some species appear to have far more RALFs than can be explained by expansion of the genome alone. These have been circled on Figure 1 and Figure S2. Interestingly, we noted that five of these species belong to the Brassicaceae. When the Brassicaceae are omitted from this analysis, the number of RALF genes correlates more strongly with the genome size (*r* = 0.31) and gene number (*r* = 0.69) for eudicots, coefficients that are much higher than before but still below those of the monocots.

### The RALF family has diverged into four major clades

**Figure 2:**
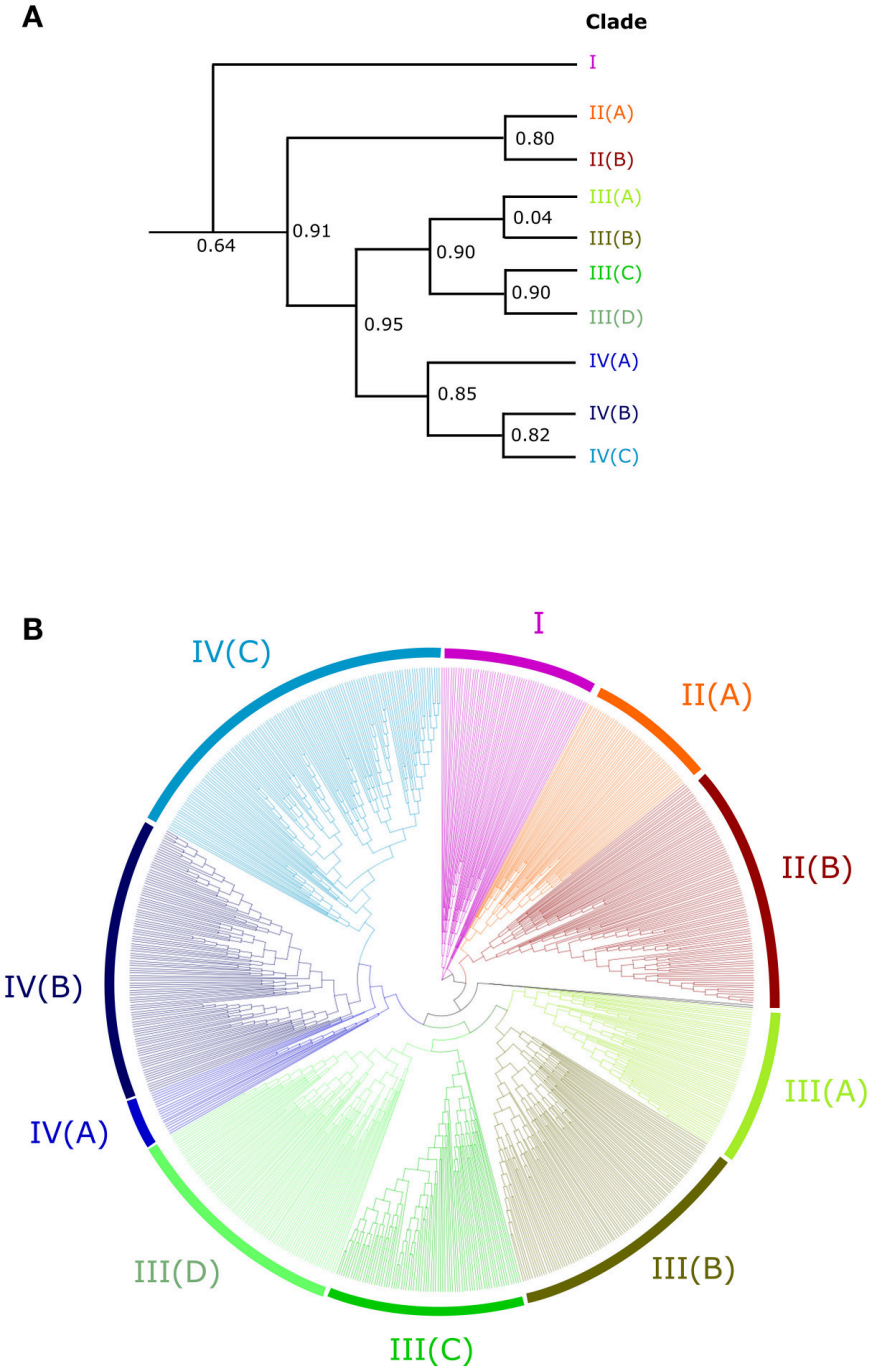
**An unrooted approximately-maximum likelihood phylogenetic tree of the 795 aligned RALF proteins from 51 plant species. (A)** A simplified cladogram representing the high-level splits of the tree, with the four major clades denoted by a number and sub-clades denoted by a letter. Local support values of these splits are provided, as calculated by the Shimodaira-Hasegawa test. **(B)** The full tree inferred from the 795 proteins. Pink, red, green and blue colors indicate clades I, II, III, and IV respectively and sub-clades are shaded appropriately.

In order to understand the evolution of the RALF family in more detail we aligned the 795 identified RALF protein sequences and inferred a phylogenetic tree The tree separates into four major clades (Figure 2) that consistently shows very high support values (>0.8; Figure 2A) Clade III is the largest of the four major clades with 320 members, followed by clade IV with 264, clade II with 151 and clade I with only 49 proteins. All four clades contain RALFs from a variety of species, including both monocots and eudicots (Table 1), suggesting that all clades evolved before the divergence of these two angiosperm lineages. However, 90% of the genes within clades I and II are from eudicot species, with the Poaceae (grasses) being entirely absent from these clades. Conversely, there is a notable overrepresentation of monocots within clade III, as 70% of the monocot RALFs are found here.

Nine RALFs were identified within the *A. trichopoda* proteome, the most basal angiosperm (Zuccolo et al., 2011). Only 4 of the 43 angiosperm species studied here contain fewer than nine RALFs, meaning that there has been a general diversification, rather than a contraction, of the RALF family within almost all lineages since the early beginnings of the angiosperms. In support of this, *A. trichopoda* contains RALF proteins belonging to clades II, III and IV, explaining why these clades are represented across both the monocots and eudicots. The early-diverging species *P. patens* and *S. moellendorffii* have fewer RALF genes than *A. trichopoda* and these are instead found within clade III. We could identify no RALF genes within the five chlorophyte species (Table 1).

Clades II, III, and IV can be further split into distinct sub-clades, with strong local support (Figure 2). Each of the nine sub-clades contains a range of species from across the angiosperms (Table 2), suggesting that these sub-clades evolved within the ancient angiosperms. This is evidenced by the spread of the 9 *A. trichopoda* RALFs across 6 of the 10 sub-clades. However, clade III(D) is almost entirely absent from the eudicots, but in monocots has expanded to become the most prevalent clade. How each of these subclades differs in terms of amino acid sequence will be considered below.

**Table 2:** **The number of RALF proteins found within each subclade per species**.

**Species**	**Total**	**II**	**II**	**III**	**III**	**III**	**III**	**IV**	**IV**	**IV**
		**A**	**B**	**A**	**B**	**C**	**D**	**A**	**B**	**C**
*Amaranthus hypochondriacus*	12	3	1	1	1	2	0	1	0	1
*Amborella trichopoda*	9	0	1	0	2	2	1	0	1	2
*Ananas comosus*	14	1	0	2	1	1	6	1	2	0
*Aquilegia coerulea*	12	0	3	1	1	1	0	0	4	1
*Arabidopsis halleri*	25	0	1	0	5	0	0	1	3	12
*Arabidopsis lyrata*	33	1	1	2	4	1	0	3	5	12
*Arabidopsis thaliana*	37	1	2	2	5	1	0	2	7	14
*Brachypodium distachyon*	10	0	0	1	0	2	6	0	0	1
*Brachypodium stacei*	11	0	0	1	0	2	7	0	0	1
*Brassica rapa*	32	3	2	3	6	3	0	1	3	5
*Capsella grandiflora*	24	1	1	2	4	1	0	0	4	8
*Capsella rubella*	33	1	1	2	5	1	0	1	4	13
*Carica papaya*	17	2	1	1	0	1	0	0	8	3
*Chlamydomonas reinhardtii*	0	0	0	0	0	0	0	0	0	0
*Citrus clementina*	13	1	3	1	1	1	2	0	0	1
*Citrus sinensis*	14	3	2	1	0	2	4	0	0	1
*Coccomyxa subellipsoidea C-169*	0	0	0	0	0	0	0	0	0	0
*Cucumis sativus*	13	2	2	1	2	1	0	0	1	2
*Eucalyptus grandis*	16	3	1	2	2	0	1	0	5	0
*Eutrema salsugineum*	35	1	1	2	7	1	0	2	4	13
*Fragaria vesca*	9	2	2	0	3	0	0	0	1	0
*Glycine max*	23	6	2	5	4	2	0	1	0	1
*Gossypium raimondii*	33	8	2	2	4	6	0	0	11	0
*Linum usitatissimum*	20	2	5	2	4	2	0	2	1	2
*Malus domestica*	33	12	2	2	7	2	0	0	6	0
*Medicago truncatula*	13	3	1	1	2	1	0	0	1	4
*Micromonas pusilla*	0	0	0	0	0	0	0	0	0	0
*Mimulus guttatus*	17	2	4	1	2	2	0	0	0	4
*Musa acuminata*	13	0	0	1	3	1	1	0	0	0
*Oryza sativa*	14	0	0	1	0	4	8	0	0	1
*Ostreococcus lucimarinus*	0	0	0	0	0	0	0	0	0	0
*Panicum hallii*	13	0	0	1	0	2	5	0	4	1
*Panicum virgatum*	31	0	0	2	0	4	16	0	7	2
*Phaseolus vulgaris*	9	3	1	1	1	1	0	1	0	0
*Physcomitrella patens*	2	0	0	0	1	1	0	0	0	0
*Populus trichocarpa*	20	2	4	2	2	3	0	0	0	5
*Prunus persica*	13	2	3	1	2	1	0	0	3	0
*Ricinus communis*	18	3	2	1	1	2	0	0	2	7
*Salix purpurea*	32	2	8	2	2	3	0	0	3	8
*Selaginella moellendorffii*	1	0	0	0	0	1	0	0	0	0
*Setaria italica*	15	0	0	1	0	2	6	1	4	1
*Setaria viridis*	15	0	0	1	0	2	6	0	5	1
*Solanum lycopersicum*	8	2	0	1	2	1	0	0	0	1
*Solanum tuberosum*	16	2	3	1	4	1	0	2	0	2
*Sorghum bicolor*	16	0	0	0	0	2	9	0	4	1
*Spirodela polyrhiza*	7	1	0	0	2	1	0	0	2	1
*Theobroma cacao*	13	3	2	1	2	2	0	0	1	2
*Vitis vinifera*	4	3	0	0	0	1	0	0	0	0
*Volvox carteri*	0	0	0	0	0	0	0	0	0	0
*Zea mays*	20	0	0	1	0	3	11	0	4	1
*Zostera marina*	7	2	0	2	2	1	0	0	0	0

### Pairwise-similarity approaches support the existence of distinct RALF clades

**Figure 3:**
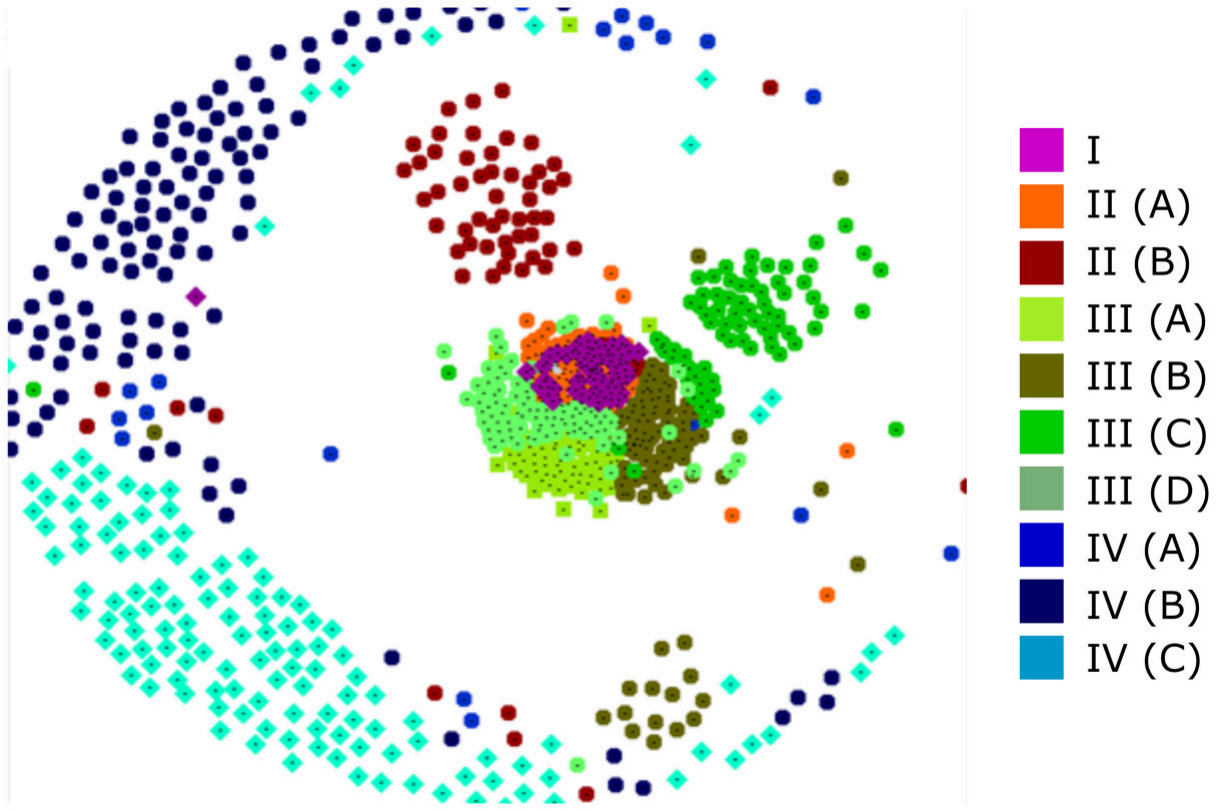
**A CLANS analysis of RALF protein similarity**. Each of the 795 full-length protein sequences is represented by a colored dot that relates to the placement of that protein within the phylogenetic tree clades and subclades. Proteins that are closer within the 2D space are considered to have more sequence similarity.

It has been established that using a large number of aligned sequences does not necessarily lead to an accurate phylogenetic tree (Philippe et al., 2011). In order to assess the accuracy of our tree and to further investigate the relationship between the various clades, we sought an alternate method of visualizing protein relationships. CLANS performs all-against-all BLAST searches upon an unaligned dataset and returns a non-deterministic 2D or 3D map in which protein similarity can be visualized (Frickey and Lupas, 2004). Figure 3 shows a typical CLANS output of the 795 full-length RALF sequences, in which each protein is represented by a colored dot. The color of each dot denotes the sub-clade that the protein fell into within the phylogenetic tree (Figure 2B) and the distance between the proteins signifies their similarity. Of note is the high level of correspondence between CLANS and the phylogenetic tree, demonstrated by scarce intermixing of proteins from different major clades within the 2D space. Hence, CLANS independently finds that RALFs generally have more sequence similarity with other proteins from the same clade than they do with members of other clades. This confirms that the major splits of the phylogenetic tree (Figure 2B) represent detectable differences in the underlying amino acid sequences. Clade IV RALFs appear to be the most diverged, as they are mostly restricted to the periphery of the CLANS map. Clade I, II and III instead fall closely together, with the central mix of clade I and II proteins suggesting that some RALFs within these clades have very similar sequences.

Furthermore, RALFs consistently cluster within the CLANS plot (Figure 3) into their respective sub-clades, signifying that these sub-clades also represent distinguishable variations in sequence. Of interest is the separation of clades II(A) and II(B), with II(A) intermixing with clade I whilst II(B) clusters slightly further away. This is in contrast with the inferred phylogenetic tree and suggests that clades I and II(A) share more sequence similarity than either does with clade II(B).

### The clades represent divergence in the mature peptide sequence

**Figure 4:**
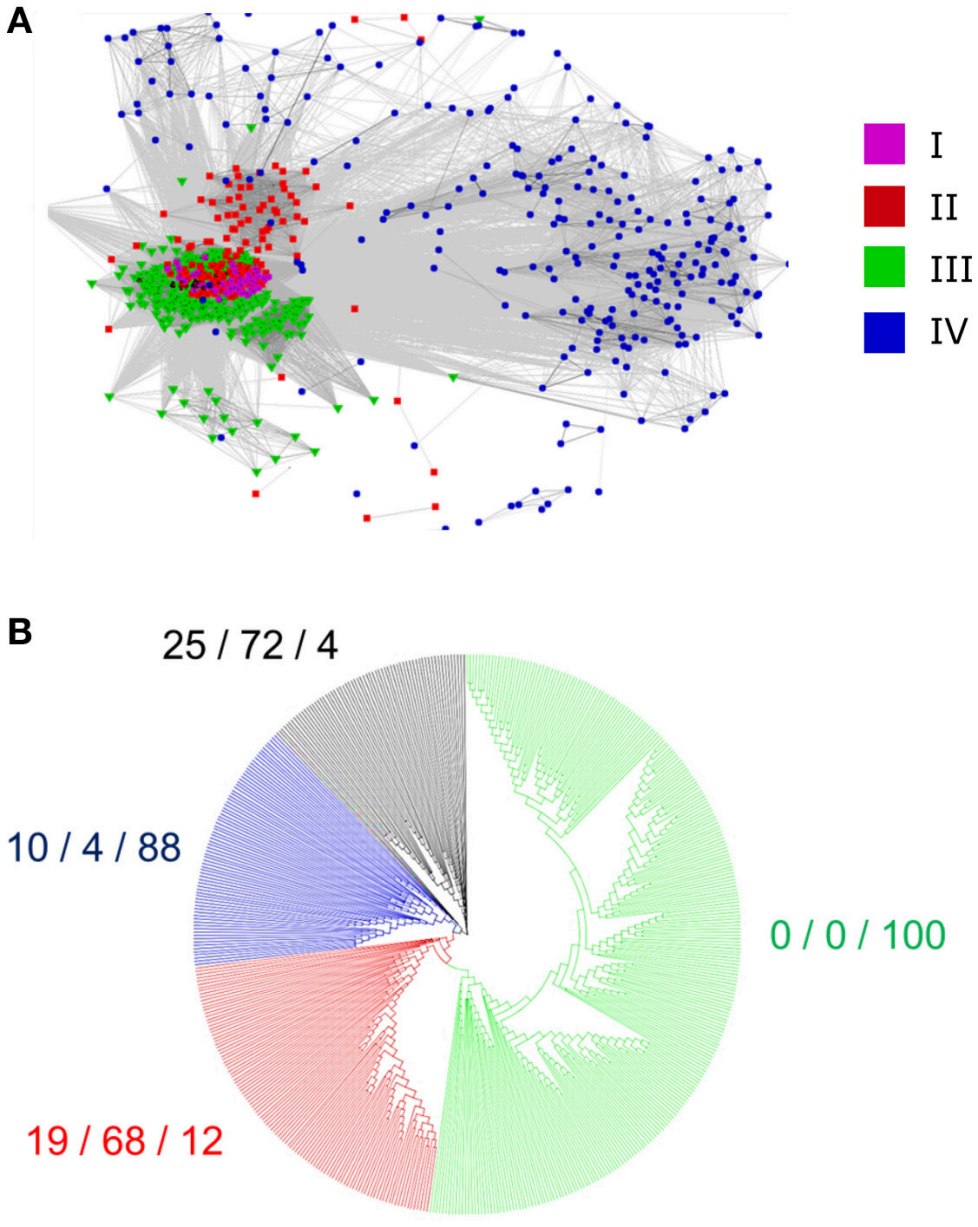
**Divergence of the mature peptide region. (A)** A CLANS sequence similarity analysis of the region downstream of the YISY motif from 795 RALF proteins. The colored dots represent the placement of each peptide within the full-length preproprotein tree (Figure 2), as denoted by the key. **(B)** An approximate-maximum likelihood tree of the mature RALF peptides placed within the full-length preproprotein clades I, II, and III. As a measure of the correspondence between the two trees, the numbers to the side of each clade show the percentage of RALFs that are placed within clade I/clade II/clade III on the full-length tree, respectively.

We questioned whether the separation of the 795 identified RALFs into four clear clades was due to variations within the mature, functional peptide sequence or because of residues outside of this region that are perhaps subject to less selection pressure. We removed the N-terminus region from the aligned sequences, leaving only the YISY motif and all downstream residues. Although the beginning of the peptide is thought to be a few residues upstream of the YISY motif (Matos et al., 2008), variation within this region makes it difficult to accurately identify the start of the peptide and hence these residues were omitted for simplicity. A CLANS analysis of this peptide region (Figure 4A) produces a very similar distribution to the full-length preproprotein CLANS, suggesting that residues within the mature peptide have sufficiently diverged alongside those outside of this region. Of note, the RALFs found within clade IV on the full-length tree again fall to the periphery of the peptide-only CLANS plot, demonstrating substantial differences within their peptide sequence compared to the other clades. Conversely, there is a tight cluster of peptides from the full-length clades I, II, and III, highlighting the similarities of these peptides. In light of this, we aligned the mature peptide region from clades I, II, and III and created an additional approximate-maximum likelihood tree to see whether the peptides from these three clades could be distinguished. The tree (Figure 4B) reliably separated the clade III mature peptides from those in clades I and II, which appear to be mostly indistinguishable. The clear overlap between the phylogenetic analyses of the full-length preproprotein and mature peptide suggests that there have been divergences across the whole length of the protein, including the functional peptide region.

### Clade-specific variations in protein sequence

**Figure 5:**
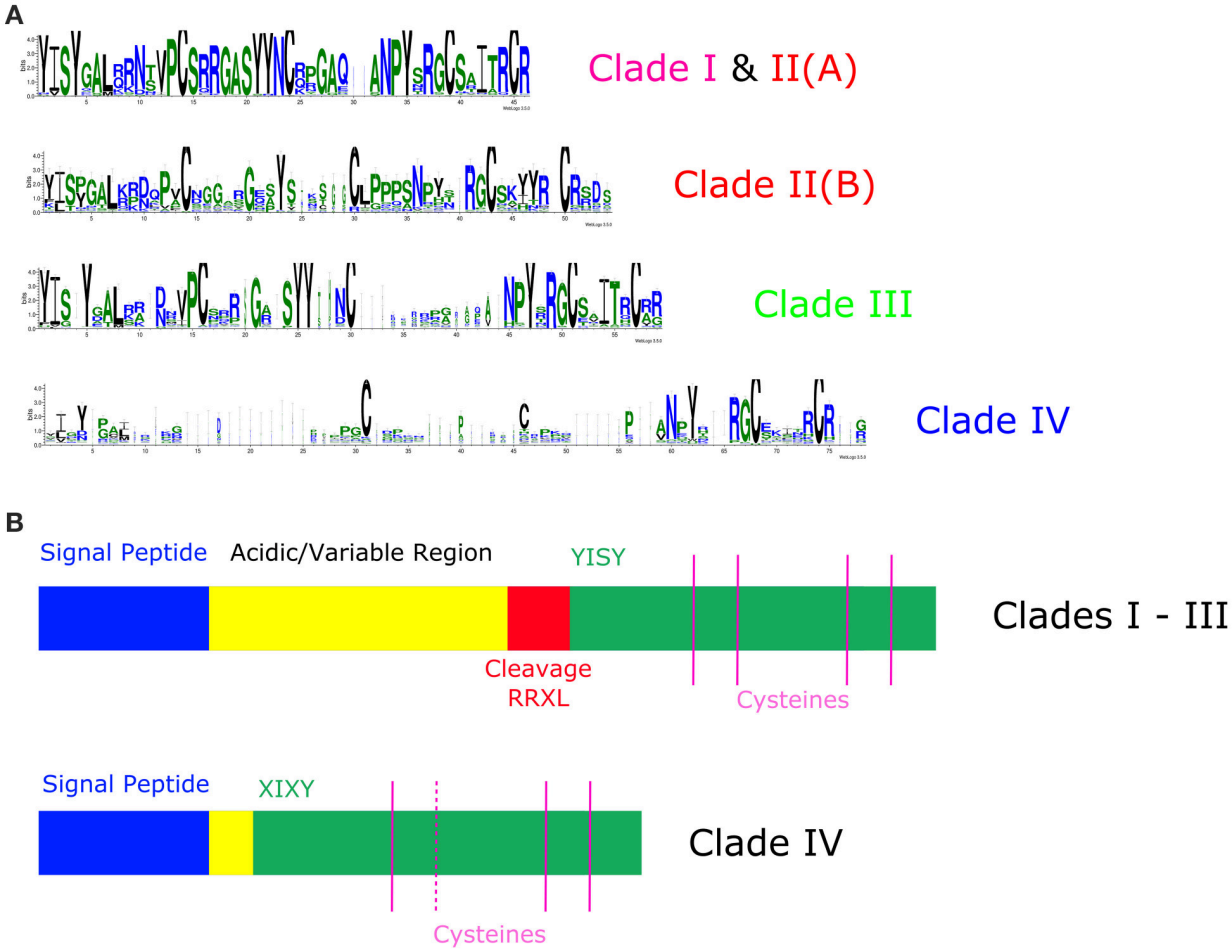
**Divergence of RALF protein sequences across the four major phylogenetic clades. (A)** WebLogo3 plots to demonstrate residue conservation within the mature peptide region of the four major clades. Clade I and II (A) are shown together as the mature peptides of these sub-clades are very similar. **(B)** A schematic representation of the motif structure of RALF proteins from clades I, II and III in comparison to the shorter RALFs of clade IV. Proteins from both clades contain an N-terminal signal peptide, but the variable and acidic region downstream of this is much shorter and frequently absent within the clade IV RALFs. The di-basic mature peptide cleavage site is absent within clade IV, suggesting an alternate cleavage mechanism. Additionally, the YISY motif thought to be required for receptor binding is highly variable within clade IV RALFs and many proteins in this clade do not contain the second of the four typically conserved cysteine residues.

As both methods of assessing protein similarity broadly agreed that the RALF family has diverged into distinct groups, we carried out a more detailed analysis of the underlying protein sequences. We aligned individual clades in order to identify any distinguishing characteristics of each. Inspection of the underlying sequences reveals that the proteins of clades I and II(A) are indeed very similar. As shown in Figure 5A, the consensus mature peptide sequence of clade II(B) is much distinct from clades I and II(A), lacking a conserved YYNC motif whilst containing additional proline residues toward the C-terminus. Such differences are consistent with the relative placement of these three clade/sub-clades within the CLANS output (Figure 3).

The RALFs that occupy clade III are also very similar to those of clade I, in agreement with their close proximity in Figure 3. However, these proteins appear to have diversified somewhat from the remarkably well-conserved RALFs that belong to clades I and II(A). There are no clear characteristics that can distinguish clade III as a whole, demonstrated by the amount of variation at many residue positions of the mature peptide (Figure 5A). Instead, each of the four subclades has seemingly diversified differently, though there is noticeable diversification even within each subclade. The most distinguishing variations are found within the mature peptide region, as can be seen in Figure S3. Obvious examples include the insertion of a proline residue at position 29 within clade III(D), and the insertion of an alanine at position nine of clade III(C) RALFs. Additionally, the CRG motif that occupies the three terminal residues of almost all clade III(D) peptides is entirely unique to that sub-clade. Notably, clades III(A/B) commonly contain an additional di-basic site upstream of the YISY motif that is mostly absent from clade III(C) and entirely absent from clade III(D). It is possible that the presence of this di-basic site within close proximity to the di-basic RALF cleavage site (RRIL; Matos et al., 2008) could affect the processing of these preproproteins.

### Clade IV RALFs are distinct and divergent

Whereas clades I, II, and III all possess the conserved YISY motif that is thought to be responsible for the binding of the peptide to its receptor (Pearce et al., 2010), remarkably this motif is rarely found within clade IV. Only 3/264 clade IV RALFs contain “YISY,” with the remainder showing a diverse range of substitutions, though many still contain an isoleucine and tyrosine at the second and fourth positions (XIXY). This lack of YISY conservation can be visualized in Figure 5A. Furthermore, many other typically-conserved residues are absent from this clade. The RRXL protease cleavage site, found upstream of YISY within the vast majority of clade I, II, and III RALFs, is almost entirely absent across the 264 clade IV proteins. This likely means that these RALFs are processed and cleaved through a different mechanism, if at all. Likewise, the acidic (glutamate/aspartate) region usually found between the signal peptide and the mature peptide is missing, with this probably impacting substantially upon the protein's structure and stability. In fact, only a minority of peptide residues are conserved within clade IV, with most residues being extremely variable (Figure 5A). The absence of these motifs and other residues results in the clade IV RALFs having a mean length of only 88 amino acids, in contrast to the other clades (Figure 5B), which contain RALF proteins with an average length of 125 amino acids. The missing regions likely explain the position of clade IV RALFs at the periphery of the CLANS 2D plot, away from the central zone occupied by the other three clades (Figure 3). The higher frequency of RALF proteins within the Brassicaceae is specifically due to an overrepresentation of clade IV RALFs, with this clade representing 56% of the RALF proteins within the Brassicaceae species analyzed, in comparison to the average representation of 34% across all 51 species.

### Clade IV has distinct physico-chemical properties and expression patterns

We questioned whether the distinctive sequence patterns of the clade IV RALFs are likely to have any significant impact upon the physico-chemical properties of the translated protein. CleverMachine (Klus et al., 2014) allows for the detection of protein properties that differ between two datasets and has been used to distinguish P-bodies and stress granules from other globular proteins (Marchese et al., 2016) and to classify homo-repeat proteins (Yu Lobanov et al., 2016). A multi-cleverMachine property prediction and comparison of the RALFs reveals that the clade IV proteins differ in a variety of physico-chemical properties from the other clades (Figure S4), such as a reduced disorder propensity. The analysis revealed that most properties could distinguish the clade IV proteins with high accuracy, as demonstrated by the typical area under the ROC curves being >0.9, a score typically considered to indicate a highly accurate test (Greiner et al., 2000).

**Figure 6:**
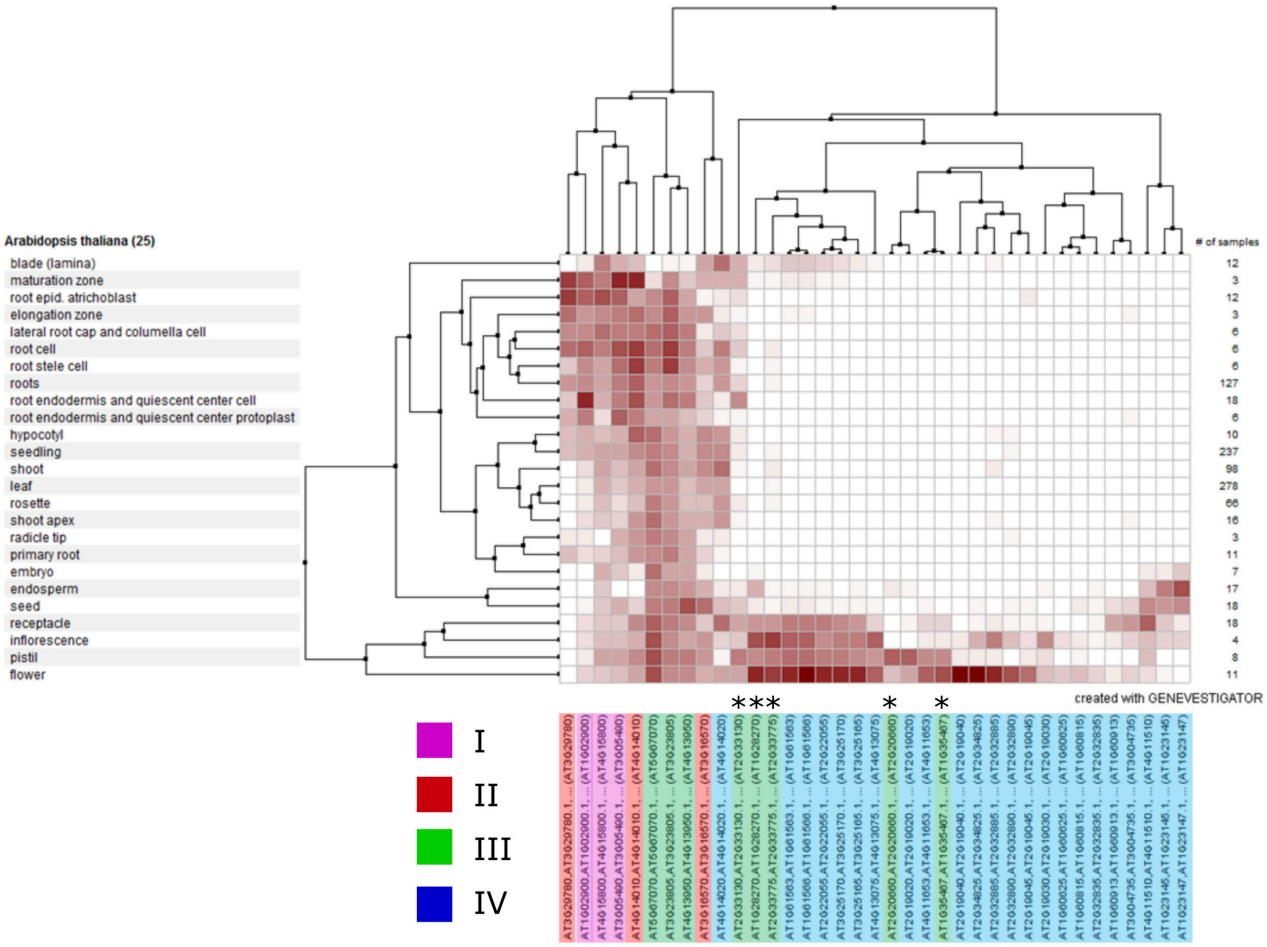
**Clustered mRNA expression values of 37 ***Arabidopsis thaliana*** RALF genes across a variety of tissues**. Each gene is colored according to its phylogenetic clade (see key). Asterisks indicate the five genes belonging to the sub-clade III(B).

To identify whether genes within clades might have a common function and hence share similar expression profiles, we analyzed the expression of each *A. thaliana* RALF gene across various publically-available RNAseq datasets using Genevestigator (Hruz et al., 2008; Figure 6). We found that the expression of clade IV RALFs is almost exclusively restricted to inflorescence tissues, with only 1 of the 23 *A. thaliana* clade IV genes (AT4G14020) showing notable levels of expression within other anatomical regions such as the root. In contrast, genes from other clades exhibit a more widespread expression profile and expression is found within the root and shoot as well as flowers, with the specific exception of the subclade III(B) which has a very similar expression pattern to the clade IV genes. This data further suggests that there have been functional diversifications between clades and genes within a clade share expression patterns. We found similar expression patterns within *Z. mays* (Figure S5), with the clade IV genes again being restricted to the inflorescence tissues.

### Diverse C-terminals of RALF peptides

Whereas most residues within the mature peptide are highly conserved, there exists a great deal of variation at the C-terminus. Although the majority of RALFs terminate with an RCRR motif, many have additional residues downstream. The composition of these residues is highly variable and each is usually restricted to a few closely related species, suggesting that these are relatively recent additions to the protein. A selection of these is shown in Figure S6 to demonstrate their variability. The longest of these are found within *L. usitatissimum*, which contains two RALF proteins with lysine-rich C-terminal tails that are over 250 residues in length. We also identified a *F. vesca* gene (gene10567-v1.0-hybrid) which is a fusion of an N-terminus LOW PSII ACCUMULATION1 (LPA1) homolog to a C-terminus RALF protein. In Arabidopsis, LPA1 is known to be involved in the assembly of photosystem II (Peng et al., 2006). Finally, whereas some CLAVATA3/ESR-related (CLE) and C-TERMINALLY ENCODED PEPTIDE (CEP) genes containing multiple peptide motifs have been reported (Oelkers et al., 2008; Sawa et al., 2008; Roberts et al., 2013), we could find no evidence of RALF genes that contain more than one RALF peptide motif.

## Discussion

In this study, we undertook a comprehensive identification and analysis of the RALF protein family across 51 plant species and more than 20 families. Previously published phylogenetic analyses of the RALF family (Cao and Shi, 2012; Sharma et al., 2016) have been limited by low species numbers (six and four, respectively), which thereby restricts their inferred evolutionary history. By including a wider variety of species in the analysis, our data should provide a more accurate representation of RALF evolution with greater resolution. We found a widespread presence of RALFs across the land plants, with the eudicots containing more RALF members on average compared to the monocots, although this is partially due to differences in genome size. This is in accordance with a previous study that found cysteine-rich small peptides such as the RALFs have generally diversified more in eudicots than monocots (Silverstein et al., 2007). Cao and Shi (2012) predicted that the most recent common ancestor of the monocots and eudicots contained two RALF proteins, based upon their identification of RALFs in *A. thaliana*, poplar, rice, and maize. However, we found that the early-diverging angiosperm *A. trichopoda* has nine RALFs, suggesting a much more widespread presence of RALF proteins within the early-diverging flowering plants than previously anticipated. We found marginally reduced estimated numbers of maize, poplar, and rice RALF proteins than Cao and Shi (2012) and Sharma et al. (2016). This is likely due to a more stringent cut-off point during our initial FIMO searches, with the benefit of our more conservative analysis being that our identified RALFs are highly likely to be genuine. Conversely, a number of the putative RALFs identified by Sharma et al. (2016) using BLAST, such as Os04g28520, appear to have very little sequence similarity to typical RALFs, to the extent that they are unlikely to be actual members of the RALF family. Additionally, a Pfam database entry exists for the RALF family (PF05498). Where the same species were analyzed by both methods, we found good correspondence between the Pfam and MEME/FIMO datasets (see Table S2). We found that a small number of highly diverged proteins were identified as RALFs by Pfam that were below the cut-off threshold of our study. Conversely, a similar number of proteins were identified by our method that Pfam did not identify, including an additional *A. thaliana* RALF. For the 29 species common to both methods, 511 RALFs were identified by Pfam in comparison to the 500 identified by MEME/FIMO, representing a minor 2% difference, suggesting that the two methods are broadly comparable in this instance. This validation of our approach allowed us to confidently apply our method across the wider range of plant species found in the 51 genomes available in Phytozome. Very recently an analogous study for the CLV3/ESR-related (CLE) family was published, in which CLE proteins were detected across all species available in Phytozome and distinct groups identified using CLANS (Goad et al., 2016). Their methods differed from ours in that Hidden-Markov Models (HMMs) were used by Goad et al. (2016) for initial peptide identification, as opposed to MEME/FIMO. However, as Pfam also uses HMMs for protein detection and our results are in good agreement with Pfam, it would seem that both methods represent valid approaches for the identification of small secretory peptides with similar accuracy.

Our inability to detect RALFs within the chlorophytes (green algae) is consistent with a previous study that could not identify small secretory peptides within these organisms (Ghorbani et al., 2015). This means that the earliest origins of the RALF family occurred after the evolution of the embryophytes (land plants). It may be that the evolution of secreted extracellular peptides allowed for the more complex, larger body plans found within the land plants. Secreted peptides are commonly associated with the local communication and control of cell proliferation, growth and differentiation (Meng et al., 2012), and the relative simplicity of the chlorophytes seemingly does not require such signaling. The recent identification of RALFs within fungi (Thynne et al., 2016) suggests that RALF pathways can be hijacked for the benefit of pathogens, further demonstrating their general importance within plant development. Whether these fungal RALFs originated through horizontal gene transfer or co-evolution is not yet clear. In contrast, the ubiquitous conservation of RALFs within every embryophyte species analyzed to date, in combination with experimentally verified roles in diverse processes such as root growth and pollen germination (reviewed by Murphy and De Smet, 2014), would suggest that the RALF family are core regulators of land plant growth and development. On the other hand, the widespread presence of RALF proteins within early-diverging lineages suggests that RALFs did not first emerge alongside any core aspects of plant development, such as pollination, with the role of RALFs within such processes probably coming later through gene duplication. Our data suggests that these duplications have occurred more rapidly within the Brassicaceae than the other species analyzed.

The inferred RALF phylogenetic trees presented by Cao and Shi (2012) and Sharma et al. (2016) frequently showed low local support values for splits, indicating that the algorithms used struggled to reliably separate the RALF family into groups. Our larger dataset allowed for an inferred tree with very high split support, which, in combination with the CLANS analysis and a detailed study of the individual protein sequences, suggests the presence of distinct RALF groups. Our phylogenetic analysis found that the RALF family has diverged into four clades. Two of these, clades I and II, can be considered the basal RALFs and proteins belonging to these clades are very well-conserved. Clade III RALFs share many similarities with those of clades I and II. Although they show some level of diversification, clades I, II, and III contain all of the features previously described to be characteristic of the RALF family, including the N-terminal signal peptide cleavage site (Pearce et al., 2001), C-terminal cysteines that form di-sulfide bridges (Pearce et al., 2001), the mature peptide YISY motif (Pearce et al., 2010), and the RRXL di-basic site (Matos et al., 2008). However, clade IV, that represents a third of the RALF family, does not contain all characteristic RALF features. Almost all clade IV RALFs lack the RRXL motif, exhibit much more variation within the YISY motif than clades I, II, and III and are much shorter and variable than the other clades. The YISY motif has been previously shown to be required for binding of AtRALF1 to its receptor (Pearce et al., 2010). Although most clade IV RALFs do possess the isoleucine residue known to be the most important within the YISY motif (Pearce et al., 2010), the widespread conservation of the four residues outside of clade IV suggests that they also have a functional role. We question whether the clade IV RALFs should be considered as a separate group from those in other clades, as such dramatic differences within their protein sequences are likely to alter their structure and processing. We therefore propose that the members of this clade are not true RALFs. A similar nomenclature has been applied to the CLE/CLE-like peptide families (Meng et al., 2012). In this case, however, RALF and RALF-like are already used by different authors to describe the same gene. Consequently, to avoid further confusion we suggest that members of clade IV should instead be referred to as RALF-related proteins. Other authors have noted that not all RALFs contain the YISY and RRXL motifs (Srivastava et al., 2009; Pearce et al., 2010; Cao and Shi, 2012; Murphy and De Smet, 2014) but here, we provide more insight into these differences within a wider phylogenetic context.

Until now, most experimental *in planta* studies assessing RALF function have focused on a minority of family members. FER is the only experimentally verified RALF receptor at this time (Haruta et al., 2014) and much work is needed to be done on the relationship between RALFs and their receptors. For instance, FER is known to control crucial fertility events such as pollen tube-ovule interactions (Huck et al., 2003), and there is also evidence for the role of RALF peptides in pollen development (Covey et al., 2010). However, we do not know how these are linked and it is not yet clear if the binding of different RALFs to FER is responsible for the extensive influence that FER has upon many aspects of development. The widespread conservation of the YISY motif across clade I-III RALFs suggests that these peptides bind to the same receptor.

In Arabidopsis, less than a third of the RALFs have been studied in any detail (Murphy and De Smet, 2014). Of these, only AtRALF8 belongs to clade IV and this is the only Arabidopsis RALF to have a proven role in regulating the response to biotic and abiotic stresses thus far (Atkinson et al., 2013). This also indicates that the clade IV RALFs are indeed functional. Furthermore, there is evidence that other receptors exist. AtRALFL4, here placed within clade III(B), actually increased in alkalinization activity in the presence of the suramin, a general inhibitor of peptide-ligand-receptor interactions, in contrast to the decreased activity of AtRALFL1, 19, 22, 23, 24, 31, 33, and 34 (Morato do Canto et al., 2014). This would suggest that some RALFs instead bind to other receptors that are not susceptible to suramin. The binding to these other receptors may or may not depend upon the YISY motif and it may be that the clade IV RALFs with their more variable motif bind to different receptors to those of other clades. As these proteins are missing the RRXL di-basic site, their cleavage and processing may also occur in an alternate manner. One other possibility is that the RALF-related peptides bind to the same receptors but act synergistically or antagonistically to the true RALFs. The presence of such antagonistic interactions between closely related peptides has recently become apparent in plants for the first time in stomatal patterning. STOMAGEN and EFP2, members of the same peptide family, competitively bind to the ERECTA receptor kinase to promote or inhibit stomatal development, respectively (Lee et al., 2015). Future experimental work could investigate whether such interactions exist between the clade I–III and the clade IV RALF peptides described here.

It is not yet known to what extent redundancy exists across the RALF family. It has been shown for other types of small peptide that genes with similar peptide motifs are more likely to have redundant and overlapping functions (Ito et al., 2006; Meng et al., 2010). As an example, CLE41, CLE42, and CLE44 are functionally redundant within vascular cell differentiation and have almost identical CLE motif sequences (Ito et al., 2006). Additionally, domain-swap experiments have demonstrated that the CLE motif itself is largely responsible for specifying the overall function of the gene, rather than the sequences outside of the motif, such as the signal peptide (Meng et al., 2010). No equivalent data exists for the RALF family, although such experiments could help to identify whether the variations that we are described here within the mature peptide region relate to their function.

## Author contributions

LC carried out the analysis. LC and ST devised the analysis and wrote the manuscript

## Funding

The author wish to acknowledge the BBSRC who was supported by an award for the BBSRC Doctoral Training Partnership programme (BB/J014478/1) awarded to the University of Manchester.

### Conflict of interest statement

The authors declare that the research was conducted in the absence of any commercial or financial relationships that could be construed as a potential conflict of interest.

## Supplementary material

Table S1 **An annotated list of the 795 identified RALFs and the sources of the genomes used in this study**.

Table S2 **A comparison of the RALF identification methods used by our study and Pfam**.
