# Supplementary material for: A Comprehensive Analysis of RALF Proteins in Green Plants Suggests There Are Two Distinct Functional Groups
Source: Front Plant Sci. 2017 Jan 24;8:37. doi: 10.3389/fpls.2017.00037 (PMC5258720; doi:10.3389/fpls.2017.00037)
Supplement: Supplementary file 3 [file DataSheet1.DOCX]

Supplementary Material

Comprehensive analysis of RALF proteins in green plants suggests there are 2 distinct functional groups.

Liam Campbell, Simon Turner*

*** Correspondence:** Corresponding Author: simon.turner@manchester.ac.uk

## Supplementary Figures


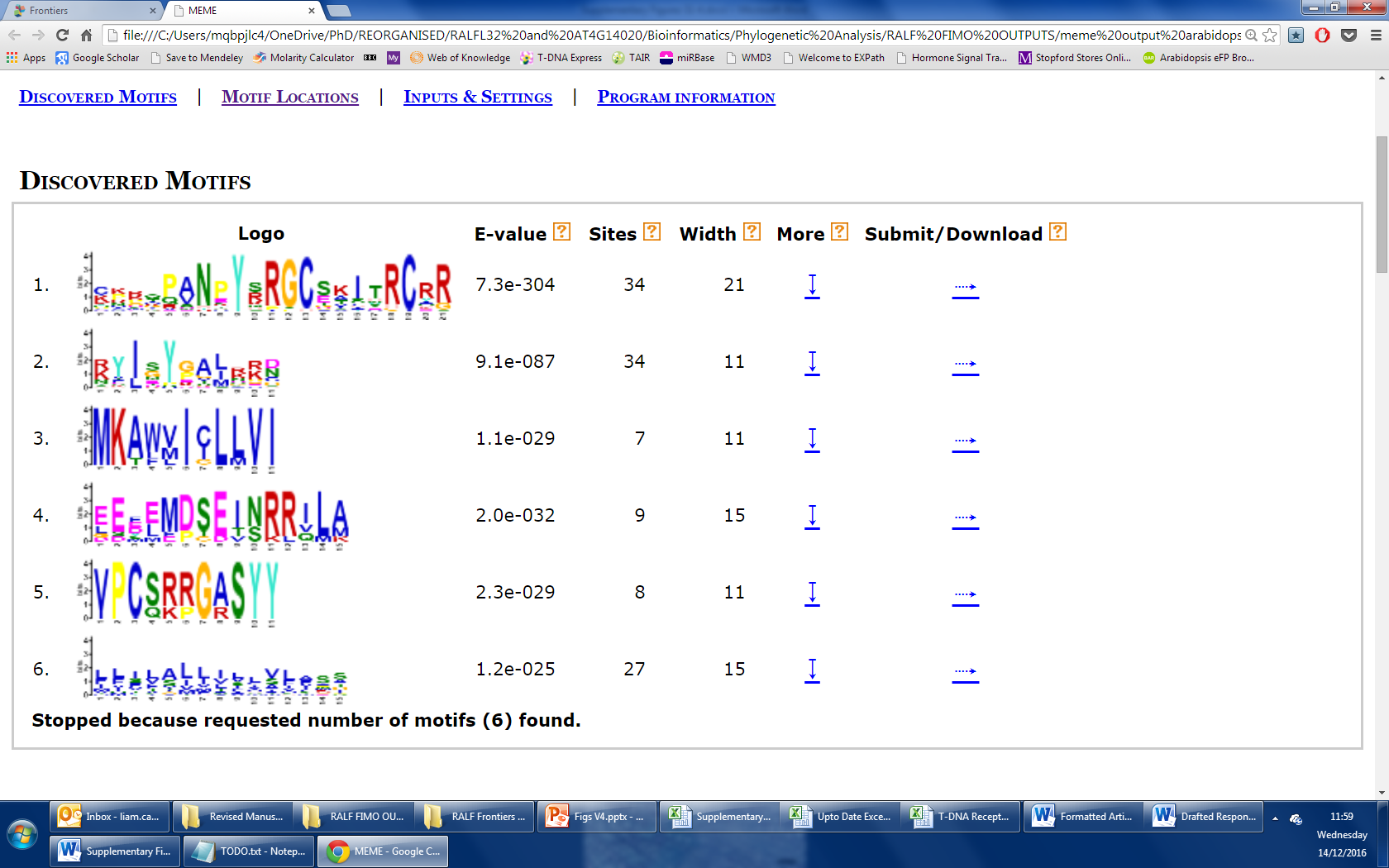


**Fig. S1 – The 6 conserved motifs identified within the *Arabidopsis thaliana* RALF family by MEME.**


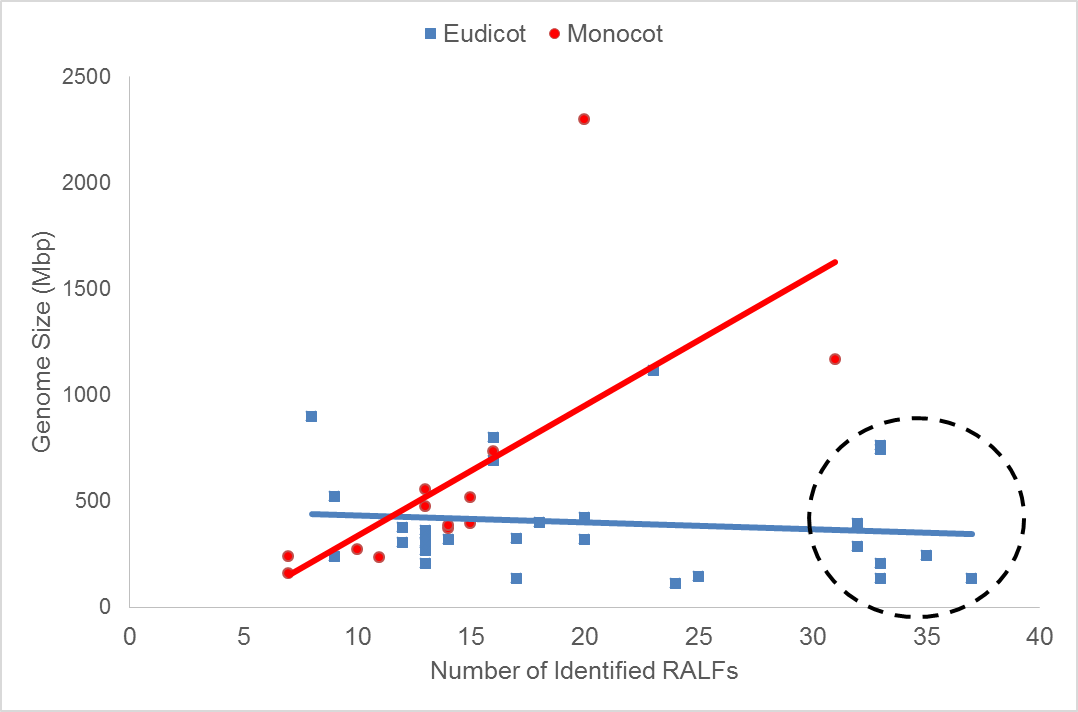


**Fig. S2 – The relationship between genome size (Mbp) and the number of identified RALFs for the monocots (red) and dicots (blue).** Species with unusually high numbers of RALFs based upon their genome content are circled.


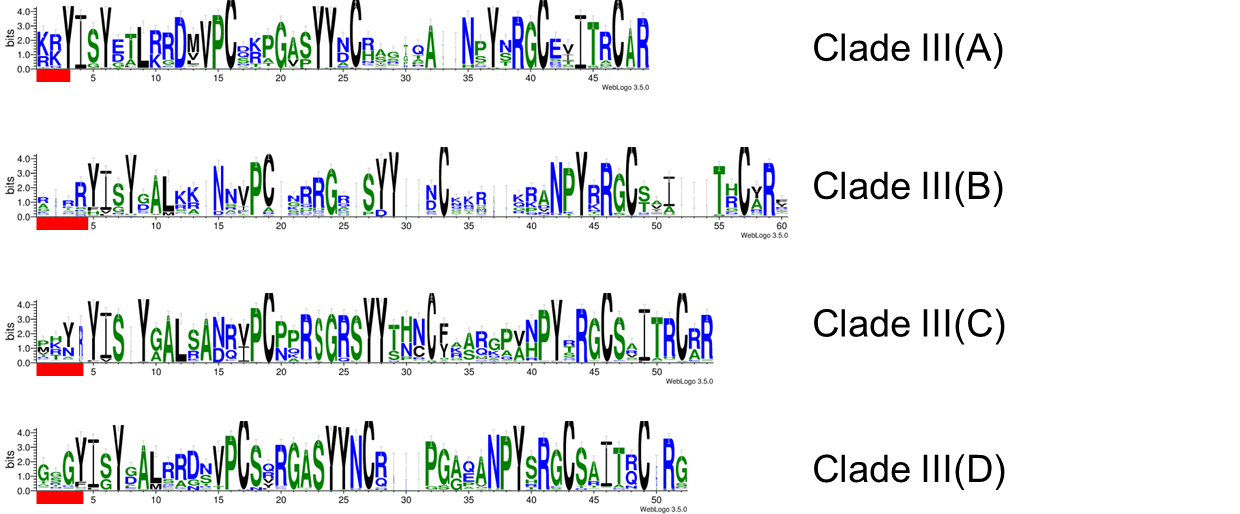


**Fig. S3 – Divergence of the mature peptide across the sub-clades of clade III.** WebLogo3 plots reveal the level of conservation at each residue. Residues immediately upstream of the YISY motif are highlighted with a red line.


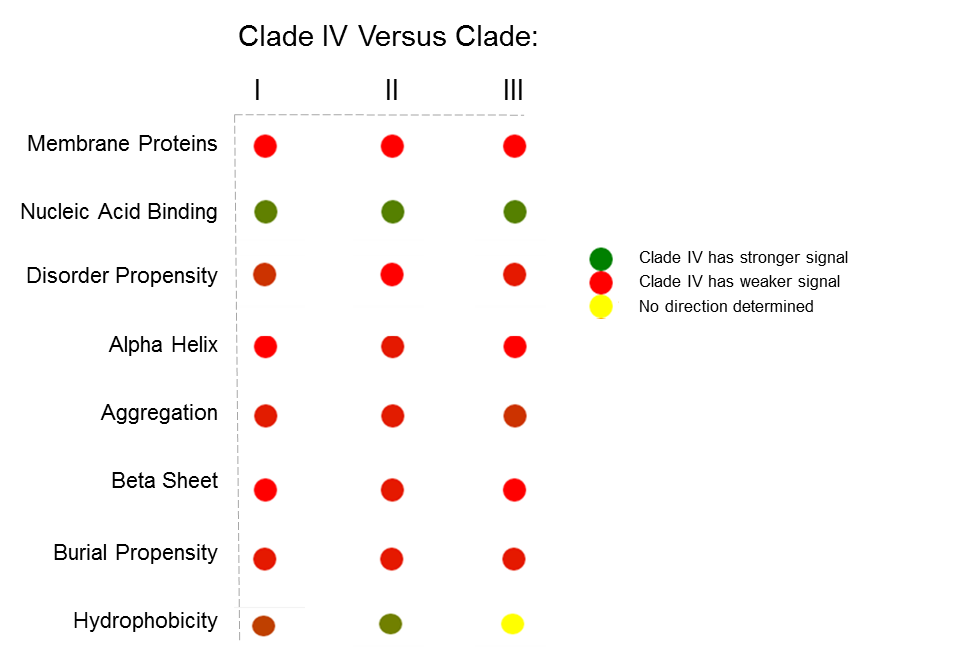


**Fig. S4 – A comparison of the physico-chemical properties of each preproprotein clade using *multi-cleverMachine*.** Ten scales representing each property were used to assess whether clade IV proteins have a stronger or weaker signal than proteins from the other three clades. Green dots indicate that clade IV has a stronger signal than the comparative clade, whereas red indicates that clade IV has a weaker signal for that property. Additional statistical data for these comparisons can be found at (<http://www.tartaglialab.com/cs_multi/confirm/1535/b7ae209a2d/>).


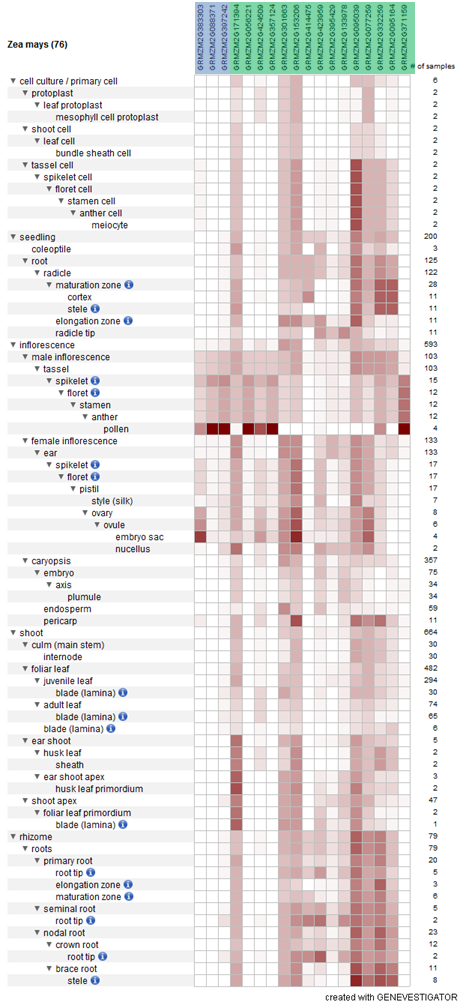


**Fig. S5 – Clustered mRNA expression values of *Zea mays* RALF genes across a variety of tissues.** Each gene is coloured according to its phylogenetic clade (green = clade III, blue = clade IV).


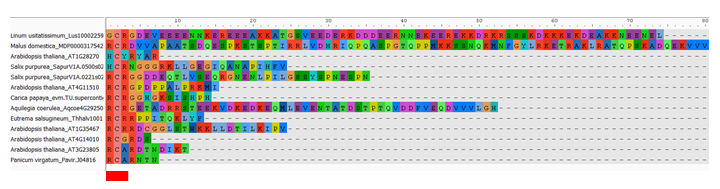


**Fig. S6 – Variation within the C-terminal region of RALF peptides.** Whereas the RALF mature peptide is typically well conserved, particularly within clades (Fig. X), the extreme C-terminal region downstream of the RCR motif (black bar) is highly variable. A small selection of these variations are shown.
